# Structural and Phylogenetic Diversity of Anaerobic Carbon-Monoxide Dehydrogenases

**DOI:** 10.3389/fmicb.2018.03353

**Published:** 2019-01-17

**Authors:** Masao Inoue, Issei Nakamoto, Kimiho Omae, Tatsuki Oguro, Hiroyuki Ogata, Takashi Yoshida, Yoshihiko Sako

**Affiliations:** ^1^Graduate School of Agriculture, Kyoto University, Kyoto, Japan; ^2^Institute for Chemical Research, Kyoto University, Kyoto, Japan

**Keywords:** carbon-monoxide dehydrogenase, genomic context, structural prediction, functional prediction, molecular evolution, horizontal gene transfer

## Abstract

Anaerobic Ni-containing carbon-monoxide dehydrogenases (Ni-CODHs) catalyze the reversible conversion between carbon monoxide and carbon dioxide as multi-enzyme complexes responsible for carbon fixation and energy conservation in anaerobic microbes. However, few biochemically characterized model enzymes exist, with most Ni-CODHs remaining functionally unknown. Here, we performed phylogenetic and structure-based Ni-CODH classification using an expanded dataset comprised of 1942 non-redundant Ni-CODHs from 1375 Ni-CODH-encoding genomes across 36 phyla. Ni-CODHs were divided into seven clades, including a novel clade. Further classification into 24 structural groups based on sequence analysis combined with structural prediction revealed diverse structural motifs for metal cluster formation and catalysis, including novel structural motifs potentially capable of forming metal clusters or binding metal ions, indicating Ni-CODH diversity and plasticity. Phylogenetic analysis illustrated that the metal clusters responsible for intermolecular electron transfer were drastically altered during evolution. Additionally, we identified novel putative Ni-CODH-associated proteins from genomic contexts other than the Wood–Ljungdahl pathway and energy converting hydrogenase system proteins. Network analysis among the structural groups of Ni-CODHs, their associated proteins and taxonomies revealed previously unrecognized gene clusters for Ni-CODHs, including uncharacterized structural groups with putative metal transporters, oxidoreductases, or transcription factors. These results suggested diversification of Ni-CODH structures adapting to their associated proteins across microbial genomes.

## Introduction

The reversible conversion between carbon monoxide (CO) and carbon dioxide (CO_2_) catalyzed by CO dehydrogenases (CODHs) constitutes a key reaction in carbon fixation and energy conservation (King and Weber, 2007; Oelgeschläger and Rother, 2008; Sokolova et al., 2009; Nitschke and Russell, 2013; Can et al., 2014). The reaction (CO_2_ + 2H^+^ + 2e^-^ <=> CO + H_2_O) serves as the initial step of carbon fixation via the carbonyl branch of the Wood-Ljungdahl pathway (WLP) in methanogens and acetogens (Nitschke and Russell, 2013; Schuchmann and Müller, 2014). This step is catalyzed by an Ni-containing CODH (Ni-CODH) subunit of the CODH/acetyl-CoA synthase (ACS) complex, which is also called the acetyl-CoA decarbonylase/synthase complex (hereafter referred to as ACS), with the CO intermediate being finally converted to acetyl-CoA by this complex (Doukov et al., 2002; Gong et al., 2008; Can et al., 2014). Additionally, organisms termed carboxydotrophs utilize CO as an energy source through this reaction due to its low redox potential (King and Weber, 2007; Oelgeschläger and Rother, 2008; Sokolova et al., 2009). Aerobic carboxydotrophs, such as *Oligotropha carboxidovorans*, use oxygen as a terminal electron acceptor from CO oxidation, with Mo- and Cu-containing CODHs belonging to the xanthine oxidase family (King and Weber, 2007; Hille et al., 2015). By contrast, anaerobic carboxydotrophs use various types of terminal electron acceptors, such as CO_2_, proton, sulfate, and ferric iron with Ni-CODHs (Oelgeschläger and Rother, 2008; Sokolova et al., 2009). For example, methanogens and acetogens use the reductive potential from CO for carbon fixation via methyl branch of WLPs (Oelgeschläger and Rother, 2008; Nitschke and Russell, 2013). In addition, hydrogenogenic carboxydotrophs, such as *Carboxydothermus hydrogenoformans* and *Rhodospirillum rubrum*, couple CO oxidation with proton reduction to produce hydrogen, in which the proton motive force might be generated with residual energy via the CODH/energy converting hydrogenase (ECH) complex (Fox et al., 1996; Soboh et al., 2002).

Ni-CODHs are divided into CooS type, which are more frequent in bacteria, and Cdh type, almost all of which are found in archaea (Techtmann et al., 2012). Structural analyses of CooS-type Ni-CODHs, such as *C. hydrogenoformans* CooSII, *R. rubrum* CooS, and *Moorella thermoacetica* AcsA, showed that the homodimeric Ni-CODH contains five metal clusters, with each subunit containing a catalytic C-cluster comprising Ni, Fe, and S along with a [4Fe-4S] cubane-type cluster (B-cluster), and a subunit interface of the dimer that accommodates an additional [4Fe-4S] cluster (D-cluster) (Dobbek et al., 2001; Drennan et al., 2001; Doukov et al., 2002). The acid–base catalysts His and Lys are located around the C-cluster (Jeoung and Dobbek, 2007; Fesseler et al., 2015). Mutations in the catalytic C-cluster and the acid–base catalysts have shown decreased activities in CO oxidation (Kim et al., 2004; Jeon et al., 2005). Intramolecular electron transfer is conducted among these three types of metal clusters, whereas intermolecular electron transfer occurs between the solvent-accessible D-cluster and ferredoxin-like proteins, such as CooF (Dobbek et al., 2001; Singer et al., 2006). Moreover, structural analysis of the *Methanosarcina barkeri* ACS α subunit showed that Cdh-type Ni-CODHs are characterized by an additional two [4Fe-4S] cubane-type clusters (E- and F-clusters) (Gong et al., 2008). These analyses suggest that the E- and F-clusters conduct intermolecular electron transfer to ferredoxin-like proteins, whereas the D-cluster conducts electron transfer to another possible electron carrier, flavin adenine dinucleotide (Gong et al., 2008).

Ni-CODHs belong to the hybrid-cluster protein (HCP) family (as *Prismane* in the Pfam database) comprising Ni-CODHs and HCPs (Finn et al., 2016). Unlike Ni-CODHs, HCP is a monomeric protein that has two metal clusters: a catalytic hybrid cluster comprising Fe, S, and O; and a [4Fe-4S] cluster, corresponding to the C- and B-clusters of Ni-CODHs (Aragão et al., 2008). Biochemical and genetic studies showed that HCPs exhibit no CODH activity, but retain the nitric oxide reductase, hydroxylamine reductase, and peroxidase activities necessary to protect cells against nitrosative and oxidative stresses (Wolfe et al., 2002; Almeida et al., 2006; Yurkiw et al., 2012; Wang et al., 2016). By contrast, Ni-CODH C-cluster variants exhibit decreased CODH activity but increased hydroxylamine reductase activity, implying catalytic similarity between these two enzymes (Heo et al., 2002; Inoue et al., 2013).

The functions of Ni-CODHs have been predicted from their genomic contexts (Matson et al., 2011; Techtmann et al., 2012). The component genes for the CODH/ACS complex are often located in gene clusters along with genes encoding their accessory factors, such as the corrinoid iron-sulfur protein and methyltransferase. Additionally, the CODH/ACS gene clusters are widely distributed in bacteria and archaea (Shin et al., 2016; Adam et al., 2018). The component genes for the CODH/ECH complex in hydrogenogenic carboxidotrophs also form gene clusters along with the genes for CooF, a CO-sensing transcriptional activator CooA, and Ni-insertion accessory proteins (Wu et al., 2005; Sant’Anna et al., 2015; Omae et al., 2017). Accordingly, Techtmann et al. (2012) classified Ni-CODHs into four functional categories: ACS, ECH, CooF, and others. Recently, a novel Ni-CODH-gene cluster containing flavin adenine dinucleotide-dependent NAD(P) oxidoreductase (FNOR) and CooF was also reported and is possibly involved in CO oxidation (Whitham et al., 2015; Geelhoed et al., 2016). Ni-CODHs are also phylogenetically classified into six distinct clades from A to F (Techtmann et al., 2012). Clade A comprises Cdh-type Ni-CODHs, and the others include CooS-type Ni-CODHs. Few Ni-CODHs in clades A, E, and F have been biochemically characterized, with Ni-CODH functions (e.g., ACS, ECH, or CooF) not necessarily related to their phylogenetic clades. Therefore, the majority of Ni-CODHs remain functionally uncharacterized (Techtmann et al., 2012). Additionally, structure-based classification of Ni-CODHs has not yet been addressed.

Current progress in next-generation sequencing techniques has expanded the available information associated with microbial genomes. Here, we expanded the datasets of Ni-CODHs and analyzed their structural variations and genomic contexts in order to reveal previously undescribed structural and functional features. Phylogenetic analysis combined with structural prediction revealed the structural plasticity of the metal clusters and active sites of Ni-CODHs. Furthermore, our genomic context analysis revealed not only novel putative Ni-CODH-associated proteins but also relationships among Ni-CODH structural features, genomic contexts, and taxonomies. Our findings suggest that Ni-CODHs are more diverse in their structures and functions than previously reported.

## Materials and Methods

### Construction of Ni-CODH Datasets

The amino acid sequences corresponding to Ni-CODHs were obtained from the National Center for Biotechnology Information (NCBI) non-redundant protein sequence database (NCBI Resource Coordinators, 2018) (as of January 4, 2018) through a BLASTp search (Camacho et al., 2009) using *C. hydrogenoformans* CooSII (WP_011343033) and the *M. barkeri* ACS α subunit (WP_011305243) as queries. Overlapping hits were excluded to construct the non-redundant dataset. Low-scoring and short-length hits (bit score <200; amino acid length <550), including HCPs and partial fragments, were excluded from the dataset (i.e., the non-redundant potential Ni-CODH dataset). Additionally, a multiple sequence alignment of the non-redundant Ni-CODH sequences was prepared using the MAFFT version 7.310 program with the E-INS-i method (Katoh and Standley, 2013). According to the alignment, sequences having no deletions in the D-, B-, and C-clusters and the catalytic residues were used for subsequent steps. To exclude Ni-CODH sequences without genomic information, 2665 Ni-CODH-encoding genomes were curated from ∼130,000 prokaryotic genomes in the NCBI assembly database in GFF3 format (NCBI Resource Coordinators, 2018) (as of January 4, 2018). These contained both genomes from cultured organisms and metagenome-assembled genomes. Moreover, genomes of same species by the NCBI taxonomy were excluded from the dataset unless these genomes encoded Ni-CODHs with different NCBI protein accession numbers in order to construct an unbiased dataset. We selected the genomes in the following order of priority: reference genomes, representative genomes, complete genomes, and draft genomes. For example, there were 1025 Ni-CODH-encoding genomes in *Clostridioides difficile*. In this case, we chose 44 genomes as representatives of this taxon. In the final dataset, the number of non-redundant Ni-CODH protein sequences was 1942 (Supplementary Table 1). The numbers of Ni-CODH-encoding genomes and Ni-CODH genes were 1375 and 2241, respectively (Supplementary Table 2). It should be noted that CODH genes annotated as pseudogenes were not included in the dataset although it has been reported that a split CODH gene functions *in vivo* by possible read-through event (Liew et al., 2016). The workflow of data retrieval is summarized in Supplementary Figure 1.

### Phylogenetic and Structural Classification of Ni-CODHs

**FIGURE 1:**
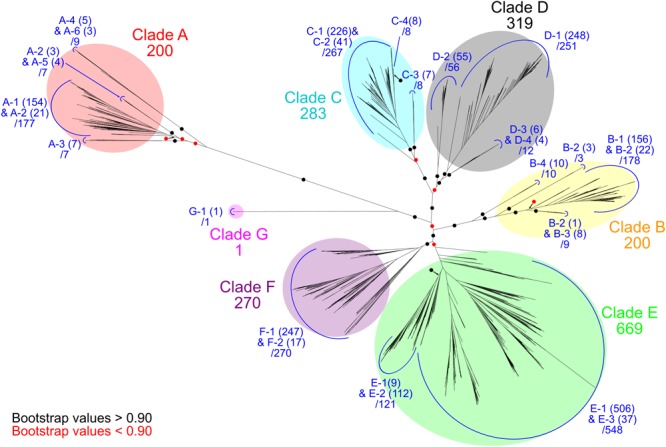
Phylogenetic relationships of Ni-CODHs. An unrooted phylogenetic tree was constructed using an alignment of 1942 Ni-CODHs. Major clades A through G are highlighted by different colors as follows: *red*, clade A; *yellow*, clade B; *cyan*, clade C; *gray*, clade D; *green*, clade E; *purple*, clade F; and *magenta*, clade G. The number of proteins in each clade is designated. Subclades are assigned by the structural groups and colored in *blue* (related to Figure 2). The number of proteins in each structural group is shown in parentheses, and the total number of proteins in each subclade is designated. The branches separating the major clades or subclades are encircled and colored according to their bootstrap values, with *black* and *red circles* indicating >0.90 and <0.90 support, respectively.

The 1942 Ni-CODH protein sequences obtained were realigned with the MAFFT version 7.390 program using the E-INS-i method (Katoh and Standley, 2013). The alignment was subsequently trimmed using the trimAl version 1.4.1 program with a gap-threshold value of 0.9 (Capella-Gutiérrez et al., 2009). A phylogenetic tree was then constructed using the FastTree version 2.1.10 program (Price et al., 2010) with an approximate-maximum-likelihood method using the WAG model. Robustness of the topology of the phylogenetic trees was evaluated by local bootstrap values based on 1000 re-samples. The six major clades (clades A–F) and the novel clade G of the tree were assigned as previously reported (Techtmann et al., 2012; Figure 1 and Supplementary Table 1). The tree was drawn using iTOL version 4.2.4 software (Letunic and Bork, 2016).

To further classify Ni-CODH protein sequences in each phylogenetic clade into structural groups, we analyzed the variation patterns of the residues comprising the D-, B-, and C-clusters and the acid–base catalysts using multiple sequence alignment (Supplementary Table 1). Additionally, novel motifs were manually searched and classified into different structural groups (see Results).

### Structural Prediction Analysis

Structural prediction of a representative protein in each structural group with unknown three-dimensional structure was performed by the homology-modeling server SWISS-MODEL (Biasini et al., 2014). The 3D structures of *M. barkeri* ACS α subunit (PDB ID: 3CF4) and *C. hydrogenoformans* CooSII (PDB ID, 4UDY) were used as templates for structural groups in clade A and in clades B to G, respectively. All models were predicted as homodimers, with the results summarized in Supplementary Table 3. All of the molecular graphics were generated using PyMOL (Schrödinger, New York, NY, United States).

### Analysis of Ni-CODH-Genomic Contexts

We searched continuous same-stranded genes [i.e., “directons” (Ermolaeva et al., 2001)], including the gene for Ni-CODH. First, Ni-CODH-encoding directons were determined according to the following criteria: the intergenic region was within 300 bp, and the number of protein-coding genes was within 15 genes upstream and downstream of the gene locus for Ni-CODH. Next, all proteins encoded in the Ni-CODH-encoding genomes were annotated with Clusters of Orthologous Groups of proteins (COGs) (Galperin et al., 2015) through RPS-BLAST searches (e-value < 10^-6^) using the NCBI Conserved Domain Database (Marchler-Bauer et al., 2017). Except for the COGs of Ni-CODHs (COG1151 and COG1152), significantly enriched COGs within the Ni-CODH-encoding directons were extracted using Fisher’s exact test (*p* < 0.05), followed by the Benjamini–Hochberg false-discovery rate control (Benjamini and Hochberg, 1995) (false-discovery rate <0.05) (Supplementary Table 4). Furthermore, the genomic contexts of genes for Ni-CODHs were curated by serially adding flanking genes annotated with the significantly enriched COGs to the Ni-CODH-encoding directon, regardless of their directions (Supplementary Table 2).

The presence or absence of the significantly enriched COGs, structural groups of Ni-CODHs, and taxonomies within each genomic context of the Ni-CODH gene were assigned to a binary matrix. A similarity scores based on the Simpson coefficient for all combinations between COGs (≥25 Ni-CODH-containing genomic loci within each COG), structural groups, and taxonomies were calculated from the binary matrix using R version 3.4.2 and its package “proxy” version 0.4–19 (Supplementary Table 5). Network analysis was performed to predict the relationships among Ni-CODH-related COGs, structural groups, and taxonomies from the similarity scores (the Simpson coefficient ≥0.4) using the R package “reshape2” version 1.4.3 and visualized using Cytoscape version 3.6.0 (Shannon et al., 2003).

## Results

### An Expanded Dataset of Ni-CODHs

We retrieved 2241 genes for Ni-CODH (1942 non-redundant protein sequences) from 1375 genomes including genomes from cultured organisms and metagenome-assembled genomes across 36 phyla and unclassified bacteria (Table 1 and Supplementary Tables 1, 2). Overall, two percent of total prokaryotic genomes encoded at least one Ni-CODH gene and 40 percent of the Ni-CODH-encoding genomes possessed more than one Ni-CODH gene. Our genome dataset was ∼eightfold larger than that from a previous study by Techtmann et al. (2012), which presented a dataset of 292 Ni-CODHs and 180 genomes across eight phyla. The newly identified 28 phyla containing Ni-CODHs were scattered around the tree of life (Hug et al., 2016). A phylogenetic tree of Ni-CODHs was constructed using 1942 non-redundant protein sequences (Figure 1). All Ni-CODHs were divided into the previously described six major clades (A–F), except for a novel clade G comprising a single protein (Figure 1 and Supplementary Table 1; Techtmann et al., 2012).

**Table 1:** The distribution of Ni-CODHs in various phyla.

Phylum	No. of genomes
Firmicutes	748 (104)*^a^*
Proteobacteria	232 (30)
Euryarchaeota	199 (36)
Chloroflexi	34
Planctomycetes	28 (2)
Nitrospirae	21
Candidatus Bathyarchaeota	13
Candidatus Omnitrophica	12
Thermodesulfobacteria	9
Aquificae	7 (4)
Lentisphaerae	6
Nitrospinae	6
Spirochaetes	6
Actinobacteria	5
Bacteroidetes	5
Elusimicrobia	5
Armatimonadetes	4
Synergistetes	4
Candidatus Kryptonia	3
Chlorobi	3 (2)
Unclassified bacteria	3
Acidobacteria	2
Candidate division WOR-3	2
Candidatus Aminicenantes	2
Candidatus Desantisbacteria	2
Candidatus Thorarchaeota	2
Crenarchaeota	2
Caldiserica	1
Candidatus Fraserbacteria	1
Candidatus Geothermarchaeota	1
Candidatus Korarchaeota	1 (1)
Candidatus Lokiarchaeota	1
Candidatus Pacearchaeota	1
Candidatus Raymondbacteria	1
Chrysiogenetes	1 (1)
Deferribacteres	1
Thaumarchaeota	1
Total	1375 (180)

*^*a*^The numbers in parentheses are from a previous study (Techtmann et al., 2012).*

### Structural and Phylogenetic Diversity of Ni-CODHs

**FIGURE 2:**
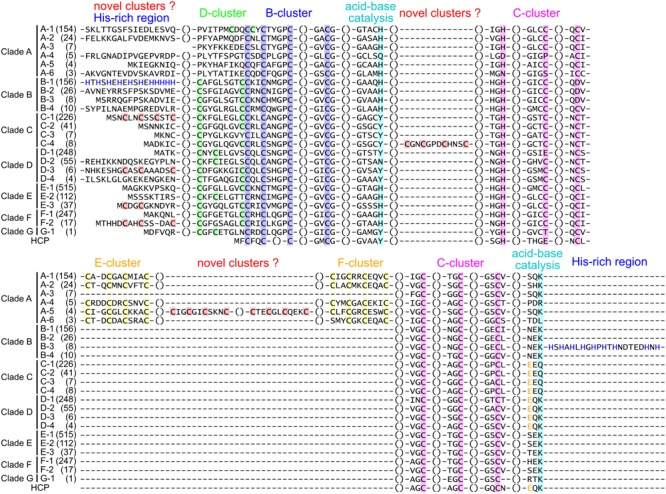
Structural groups of Ni-CODHs. The representative protein sequences of each structural group of Ni-CODHs and HCPs are represented by multiple sequence alignments. The alignment of the N- and C-terminal extensions and the insertions was manually modified for representation. The number of proteins in each structural group is shown in parentheses. The Cys residues of the metal clusters and catalytic residues are highlighted by different color backgrounds as follows: *green*, D-cluster; *blue*, B-cluster; *magenta*, C-cluster; *yellow*, E- and F-clusters; *cyan*, acid-base catalysts; and *red*, putative novel clusters. The His residues in the His-rich regions of structural groups B-1 and B-4 are shown in *blue letters*, and the Glu residues at the equivalent position of Glu494 in the catalytic hybrid cluster of *D. vulgaris* HCP are shown in *yellow letters* (Aragão et al., 2008). The NCBI accession numbers of the representative sequences are as follows: A-1, WP_011305243; A-2, WP_010878596; A-3, OGW06734; A-4, OIP92259; A-5, ODS42986; A-6, OIP30420; B-1, WP_026514536; B-2, WP_015485077; B-3, WP_012645460; B-4, WP_011393470; C-1, WP_039226206; C-2, WP_013237576; C-3, WP_010870233; C-4, WP_044921150; D-1, WP_011342982; D-2, WP_015926279; D-3, WP_079933214; D-4, WP_096205957; E-1, WP_012571978; E-2, WP_010939375; E-3, WP_088535808; F-1, WP_011343033; F-2, WP_011389181; G-1, OGP75751; and HCP, WP_010939296.

According to multiple sequence alignment analysis, we classified each phylogenetic clade into structural groups based on the variation patterns of the catalytically important residues (Figure 1, Table 2, and Supplementary Table 1). The multiple sequence alignment of the representative sequence in each structural group is shown in Figure 2. The representative sequences were chosen by the following order of priority: 3D structures are known; genes or proteins are previously described; and RefSeq sequences are available. The sequence motifs of the D-clusters were classified into three types as follows: type I, -Cys-X-X-Cys-Cys-; type II, -Cys-(X)_7-13_-Cys-Cys-; and type III, -Cys-X-X-Cys-(X)_5_-Cys- (Figure 2, Table 2, and Supplementary Table 1). Additionally, we found that there were D-cluster regions with only one or no Cys residues (see below), as previously reported (Lindahl, 2002). The four Cys residues in the B-cluster and the C-terminal three Cys residues in the C-clusters were highly conserved in all Ni-CODHs with a few exceptions, whereas the other residues in the C-cluster, the acid–base catalysts, and the E- and F-clusters were variable (Figure 2, Table 2, and Supplementary Table 1). Moreover, we found novel Cys motifs predicted to form metal clusters at the N-terminus and insertions, as well as His-rich extensions predicted to bind metal ions at N- or C-termini. Ni-CODHs fused to other proteins were also manually searched and classified (Supplementary Table 1). Based on these variations in primary structures, 1921 of the 1942 Ni-CODHs were classified into 24 structural groups, with these groups containing two or fewer proteins grouped into “others,” except for structural group G-1 (Figure 2, Table 2, and Supplementary Table 1). The phylogenetic tree showed that some of these structural groups formed subclades, whereas others did not, implying the evolutionary plasticity of Ni-CODHs (Figure 1).

**Table 2:** The comparison of structural signatures among structural groups of Ni-CODHs.

Structural group	D-cluster	C-cluster						Acid-base		E- & F-clusters	Novel structural motifs
		His	Cys1	Cys2	Cys3	Cys4	Cys5	His	Lys		
A-1	type I	H	C	C	C	C	C	H	K	+	
A-2	–	H	C	C	C	C	C	H	K	+	
A-3	–	H	C	C	C	C	C	H	K	–	
A-3	–	H	C	C	C	C	C	H	K	–	
A-4	–	D	P	C	C	C	C	Q	R	+	
A-5	–	H	C	C	C	C	C	H	K	+	Two Cys × 4 motifs between E- and F-clusters
A-6	–	N	S	C	C	C	C	H	L	+*^b^*	
B-1	type II	H	C	D/E	C	C	C	H	K	–	His-rich region at the N-terminus
B-2	type II	H	C	D	C	C	C	H	K	–	
B-3	type II	H	C	D	C	C	C	H	K	–	His-rich region at the C-terminus
B-4	type II	H	C	C	C	C	C	H	K	–	
C-1	type II	H	C	C	C	C	C	Y	Q	–	A Cys × 2 to 4 motif at the N-terminus
C-2	type II	H	C	C	C	C	C	Y	Q	–	
C-3	–*^a^*	H	C	C	C	C	C	Y	Q	–	
C-4	type II	H	C	C	C	C	C	Y	Q	–	A Cys × 4 motif between the Tyr and His residues
D-1	type III	H	E	C	C	C	C	Y	K	–	
D-2	type III	H	C	C	C	C	C	N	K	–	
D-3	type II	H	C	C	C	C	C	Y	K	–	A Cys × 3 motif at the N-terminus
D-4	type II	H	C	C	C	C	C	Y	K	–	
E-1	type II	H	C	C	C	C	C	H	K	–	
E-2	type III	H	C	C	C	C	C	H	K	–	
E-3	type II	H	C	C	C	C	C	H	K	–	A Cys × 2 to 3 motif at the N-terminus
F-1	type II	H	C	C	C	C	C	H	K	–	
F-2	type II	H	C	C	C	C	C	H	K	–	A Cys × 2 to 3 motif at the N-terminus
G-1	type III	H	T	C	C	C	C	Y	K	–	

*^*a*^One Cys residue was substituted to other amino acids.*^*b*^*One Cys residue in the F-cluster was substituted to other amino acids.*

Clade A Ni-CODHs were divided into six structural groups (A-1–6) (Figure 2, Table 2, and Supplementary Table 1). Structural group A-1 was typical of clade A Ni-CODHs, such as the *M. barkeri* ACS α subunit characterized by complete active-site motifs, the type I D-cluster, and the E- and F- clusters (Gong et al., 2008). Conversely, structural groups A-2 through A-6 had no Cys motifs in the region of the D-cluster. Among these, structural group A-3 showed no Cys signatures in the region of E- and F-clusters, whereas structural group A-5 showed an additional two Cys × 4 signatures between the E-and F-clusters. Structural groups A-4 and A-6 featured incomplete active-site signatures in both the C-cluster and acid–base catalysts. Additionally, structural group A-6 had an incomplete F-cluster.

Clade B Ni-CODHs harboring type II D-clusters and complete acid–base catalysts were divided into four structural groups (B-1–4) based on variations in the second Cys residue of the C-cluster and the presence or absence of His-rich extensions (Figure 2, Table 2, and Supplementary Table 1). Structural group B-1 had an Asp or Glu residue in place of the second Cys residue of the C-cluster and the N-terminal His-rich region. In structural groups B-2 and B-3, the second Cys residue of the C-cluster was replaced by an Asp residue, and only structural group B-3 had the C-terminal His-rich region. These His-rich regions containing from 4 to 48 His residues were characterized by the repeat of the -His-X-His- motif and similar to the C-terminal extension of CooJ and the N-terminal extension of HypB, which constitute Ni-chaperones associated with Ni-CODHs and hydrogenases, respectively (Watt and Ludden, 1998; Olson and Maier, 2000).

In all four structural groups (C-1–4) of clade C Ni-CODHs, the residues for the C-cluster were completely conserved, and the acid–base catalysts His and Lys were replaced by Tyr and Gln, respectively (Figure 2, Table 2, and Supplementary Table 1). Structural groups C-1, C-2, and C-4 had type II D-clusters, whereas structural group C-3 had only one Cys residue in the region of the D-cluster. We identified novel Cys motifs in structural groups C-1 and C-4, with structural group C-1 having an additional Cys motif containing two to four Cys residues at the N-terminal region, whereas structural group C-4 showed a Cys × 4 signature between the Tyr residue and the His residue in the C-cluster.

Clade D Ni-CODHs were divided into four structural groups (D-1–4) (Figure 2, Table 2, and Supplementary Table 1). Structural groups D-1 and D-2 had the type III D-cluster, whereas structural groups D-3 and D-4 had the type II D-cluster. In structural group D-1, the first Cys residue of the C-cluster was replaced by a Glu residue. Additionally, structural groups D-1, D-3, and D-4 had a Tyr residue at the position of the acid–base catalyst His, whereas structural group D-2 had an Asn residue. Structural group D-3 had an additional Cys motif containing three Cys residues at the N-terminal region.

All structural groups of clades E and F Ni-CODHs (E-1–3 and F-1–2, respectively) displayed conservation of the complete active-site motifs (Figure 2, Table 2, and Supplementary Table 1). Structural groups E-1, E-3, F-1, and F-2 had the type II D-cluster, whereas structural group E-2 had the type III D-cluster. Structural groups E-3 and F-2 had an additional Cys motif containing two or three Cys residues at the N-terminal region. Most of the well-characterized Ni-CODHs were classified into the structural groups of clades E and F: structural group E-1 contained *Thermococcus onnuriensis* CooSI, whereas structural group E-2 contained *D. vulgaris* CooS. Structural group F-1 contained *C. hydrogenoformans* CooSII and *M. thermoacetica* AcsA, whereas structural group F-2 contained *R. rubrum* CooS (Drennan et al., 2001; Doukov et al., 2002; Jeoung and Dobbek, 2007; Hadj-Saïd et al., 2015; Schut et al., 2016).

The novel clade G (namely structural group G-1) comprised only a single protein and was the most deeply branched among CooS-type Ni-CODHs (Figure 1). Structural group G-1 had the type III D-cluster, with the acid–base catalyst His and the first Cys residue replaced by Tyr and Thr, respectively (Figure 2, Table 2, and Supplementary Table 1). It should be noted that the sequence was derived from the high quality metagenomic assembly (completeness >80% by CheckM) (Parks et al., 2015; Anantharaman et al., 2016).

Some of these atypical structural motifs emerged across clades (Figure 2, Table 2, and Supplementary Table 1). The type III D-cluster was found in structural groups D-1, D-2, E-2, and G-1. The Tyr residue replacing to the acid–base catalyst His was conserved through structural groups of clades C, D, and G except for structural group D-2. The N-terminal additional Cys motifs were found in structural groups C-1, D-3, E-3, and F-2.

### Structural Prediction Analysis Reveals the Plasticity of the D-Clusters and Active Sites

**FIGURE 3:**
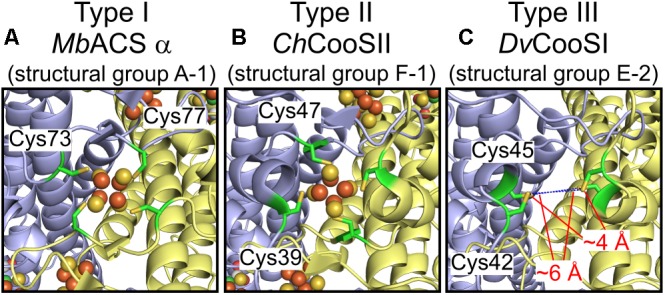
Structural plasticity of the D-clusters. **(A)** The structure of the type I D-cluster of the *M. barkeri* ACS α subunit (structural group A-1; PDB ID: 3CF4). **(B)** The structure of the type II D-cluster of *Carboxydothermus hydrogenoformans* CooSII (structural group F-1; PDB ID: 4UDX). **(C)** The predicted structure of *D. vulgaris* CooS (structural group E-2; NCBI protein accession number: WP_010939375) around the type III D-cluster. All structures are shown in cartoon representation, and each subunit is shown in different colors. The four Cys residues forming the D-cluster are represented as *green sticks*. The Fe and S atoms of the D-clusters are colored in *brown* and *yellow*, respectively. The distances between the Cys residues of the type III D-cluster are indicated.

Structural information of Ni-CODHs is limited to structural groups A-1, F-1, and F-2 Ni-CODHs. To obtain structural information regarding variations in the structural motifs, we performed structural prediction by homology modeling (Supplementary Table 3). First, we compared the conformation of three types of D-clusters. Structural analyses of the *M. barkeri* ACS α subunit (structural group A-1; PDB ID: 3CF4) containing a type I D-cluster and *C. hydrogenoformans* CooSII (structural group F-1; PDB ID: 4UDX) containing a type II D-cluster demonstrated that these two D-clusters exhibit different conformations, albeit complete formation of iron-sulfur clusters (Figures 3A,B; Gong et al., 2008; Fesseler et al., 2015). The predicted structure of *D. vulgaris* CooS (structural group E-2) with the type III D-cluster showed that the two Cys residues from each subunit faced each other, and that the distance between these four Cys residues was between 4 and 6 Å (Figure 3C). These data were consistent with a recent study of the metal-cluster formation of *D. vulgaris* CooS (Hadj-Saïd et al., 2015) and suggested that the Cys motif of the type III D-cluster could form an iron-sulfur cluster in a manner different from other types of D-clusters.

**FIGURE 4:**
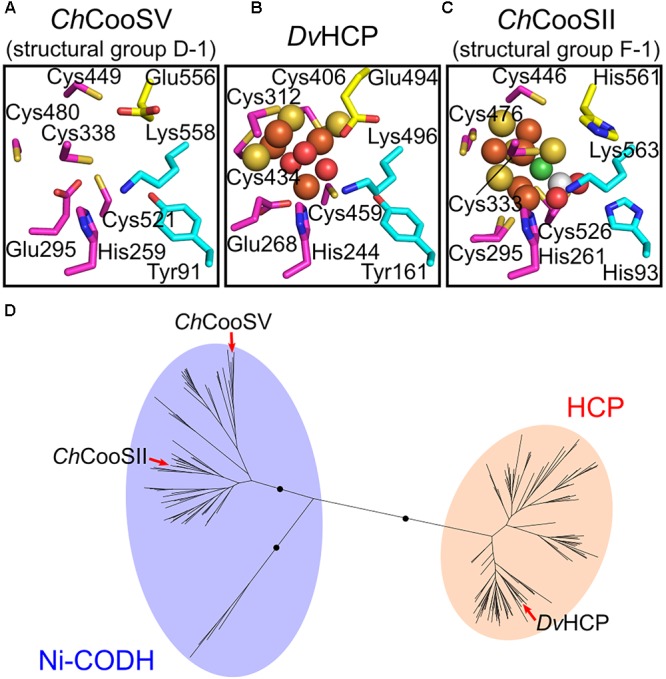
Structural and phylogenetic relationships of the catalytic sites between structural group D-1 Ni-CODHs and HCPs. **(A)** The predicted structure of the catalytic site of *C. hydrogenoformans* CooSV (structural group D-2; NCBI protein accession number: WP_011342982). **(B)** The structure of the catalytic site of *D. vulgaris* HCP (PDB ID: 1W9M). **(C)** The structure of the catalytic site of *C. hydrogenoformans* CooSII (structural group F-1; PDB ID: 4UDX). The residues forming the catalytic sites are represented in *stick forms* and colored as follows (related to Figure 2): *magenta*, the C-clusters and hybrid cluster; *cyan*, acid–base catalysts; and *yellow*, the Glu residue unique to the hybrid cluster of HCPs and the corresponding residues of *C. hydrogenoformans* CooSV and CooSII. The Ni, Fe, S, and O atoms of the metal clusters are colored in *green, brown, yellow*, and *red*, respectively. The C and O atoms of CO_2_ are colored in *white* and *red*, respectively. **(D)** The phylogenetic tree of the HCP family from the Pfam seed dataset (PF03063) (Finn et al., 2016). The branches of *C. hydrogenoformans* CooSII and CooSV and *D. vulgaris* HCP are indicated by *red arrows*.

Next, we focused on the structural variations of the active sites. Notably, the sequence alignment of Ni-CODHs with HCPs showed that the eight residues comprising the catalytic site of structural group D-1 were identical to those of HCPs (Figure 2; Aragão et al., 2008). Moreover, one additional residue forming the catalytic metal cluster of HCPs [Glu (two residues before the acid–base catalyst Lys)] was conserved in structural group D-1 Ni-CODHs. Structural prediction of *C. hydrogenoformans* CooSV showed that conformation of the active-site residues of structural group D-1 Ni-CODH resembled that of *D. vulgaris* HCP (PDB ID: 1W9M) rather than *C. hydrogenoformans* CooSII (Figures 4A–C). The root mean square deviation of these nine residues between *C. hydrogenoformans* CooSV and *D. vulgaris* HCP was 1.2 Å, although the phylogenetic tree of the HCP family showed structural group D-1 Ni-CODHs as being distant from HCPs (Figure 4D).

### Novel Ni-CODH-Associated Proteins Predicted According to Genomic Contexts

To investigate how such structural features of Ni-CODHs are related to their biological function and to predict the functional properties of each Ni-CODH, we focused on genomic contexts for Ni-CODHs. We classified all proteins encoded in the Ni-CODH-encoding genomes into COGs, followed by enrichment analysis to curate significantly abundant COGs in the Ni-CODH-encoding directons (Supplementary Table 4). The significantly enriched COGs (false-discovery rate <0.05) included a series of previously described Ni-CODH-associated proteins, such as CooF (COG0437 and COG1142), CooC (COG3640), CooA (COG0664), FNOR (COG1251), and components of WLP and ECH (Supplementary Table 4). Additionally, our analysis identified novel putative Ni-CODH-associated proteins, including various types of oxidoreductases, ABC transporters, and transcription factors, as detailed in subsequent sections. Based on these data, we determined the genomic contexts of genes for Ni-CODH by adding flanking genes annotated with these COGs to the Ni-CODH-encoding directon (Supplementary Table 2).

### Relationships Between Structural Groups and Genomic Contexts for Ni-CODHs

**FIGURE 5:**
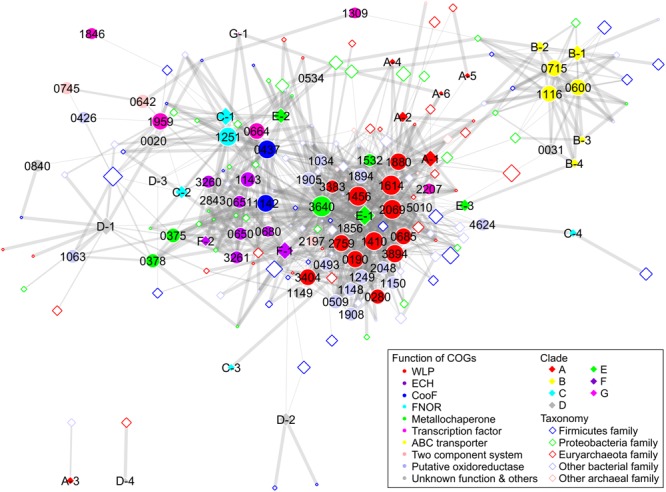
Network analysis of Ni-CODH-related proteins, structural groups of Ni-CODHs, and taxonomies focusing on the genomic contexts. The network is represented by an unweighted force-directed layout and manually modified to avoid the overlapping nodes. The width of the edges is scaled proportionally according to the Simpson coefficient. The size of the nodes is scaled proportionally according to the log of the number of gene loci of Ni-CODHs with each COG, structural group, and taxonomy. The COGs, structural groups of Ni-CODHs, and family level taxonomies are shown by filled circles, filled diamonds, and open diamonds, respectively, and colored according to their functions, clades, and phyla as *inset*, respectively. The taxonomy omitted network is shown in Supplementary Figure 3 in order to explain the functional groups of the Ni-CODH-related proteins.

To elucidate the structural and functional relationships of Ni-CODHs, we performed comprehensive network analysis using similarity scores among structural groups of Ni-CODHs, Ni-CODH-associated proteins, and taxonomies of Ni-CODH-encoding genomes based on the genomic contexts of 2457 genes for Ni-CODH (Figure 5, Supplementary Figures 2, 3, and Supplementary Table 5). The network precisely presented functional gene clusters encoding Ni-CODHs, such as WLP, ECH, and FNOR (Techtmann et al., 2012; Geelhoed et al., 2016; Shin et al., 2016).

Structural groups A-1, A-2, A-4, A-5, A-6, E-1, E-3, and F-1 Ni-CODHs were associated with the largest group comprising proteins involved in both methyl and carbonyl branches of WLP, including the ACS catalytic β subunit (COG1614) and other subunits (Figure 5 and Supplementary Figures 2, 3). This group also contained various types of oxidoreductases, such as glycine-cleavage system components (COG0509 and COG1249), heterodisulfide reductase-like proteins (COG1148, COG1150, COG1908, and COG2048), and NADH:ubiquinone oxidoreductase-like proteins (COG1034, COG1894, and COG1905). Metallochaperones were also found in the WLP group, including CooC and the recently characterized Ni-insertion accessory protein CooT (COG1532) (Timm et al., 2017). Structural group E-2 Ni-CODHs were also associated with CooC, which was in turn related to the ECH group, as well as the WLP group.

Structural groups F-1 and F-2 Ni-CODHs were associated with ECH-related proteins, including [NiFe] hydrogenase catalytic subunits (COG3261 and COG3260), accessory subunits (COG0650, COG0651, and COG1143), and their maturation protease (COG0680) (Figure 5 and Supplementary Figures 2, 3). The metallochaperones HypA (COG0375) and HypB (COG0378), CooA, and CooF (COG1142) were also classified into the ECH group.

Structural groups C-1 and C-2 Ni-CODHs were grouped with FNOR, CooF (COG0437), an NsrR/IscR-like transcriptional regulator (COG1959), and functionally unknown proteins (Figure 5 and Supplementary Figures 2, 3). CooX (COG1143), a ferredoxin-like accessory protein of the ECH complex (Soboh et al., 2002), was clustered into the FNOR group, as well as the ECH group, suggesting potential involvement in Ni-CODH/FNOR function. Notably, two orthologous groups of CooF (COG0437 and COG1142) were mainly categorized into different functional groups: COG0437 was associated with FNOR, whereas COG1142 was associated with ECH. Structural groups D-3 and G-1 Ni-CODHs were also related to FNOR and COG1142, suggesting the functional plasticity of these CooF-like proteins.

Furthermore, the network also revealed novel putative gene clusters for Ni-CODH (Figure 5 and Supplementary Figures 2, 3). All structural groups of clade B Ni-CODHs were grouped with the NitT/TauT family ABC transporter components (COG0600, COG0715, and COG1116). Structural group D-1 Ni-CODHs were associated with a functionally unknown zinc-binding dehydrogenase family protein (COG1063) along with the ECH-related metallochaperones HypA and HypB (Riveros-Rosas et al., 2003; Watanabe et al., 2015). Structural group C-4 was associated with a [FeFe] hydrogenase catalytic subunit (COG4624). A histidine kinase (COG0642) and a response regulator (COG0745), as a two-component system, were not related to any structural groups of Ni-CODHs, suggesting co-existence with this system and various structural groups of Ni-CODHs. The genomic context analysis suggested that these proteins likely exhibit Ni-CODH-associated functions; however, the functional relationships between these proteins and Ni-CODHs remain to be elucidated.

### Ni-CODH Genomic Contexts Are Scattered Across Microbial Genomes

Our network analysis also revealed taxonomic profiles of Ni-CODHs (Figure 5). Regardless of their phyla or domains, the family level taxa were scattered across the network and associated with various types of Ni-CODHs and their related proteins. A previous study speculated that clade A Ni-CODHs are specific to archaea with one exception, whereas clades B and F are specific to bacteria (Techtmann et al., 2012). In the present study, however, all clades of Ni-CODHs were found in both domains (Figure 5 and Supplementary Tables 2, 5). Among these, structural group A-1 Ni-CODHs were mainly found in archaea, whereas structural groups B-1, C-2, and F-1 Ni-CODHs were mainly found in bacteria, with a few exceptions. Structural groups A-2, A-5, C-3, and D-4 were found only in archaea, whereas structural groups A-3, A-4, A-6, B-2, B-3, B-4, C-1, C-4, D-2, D-3, E-3, F-2, and G-1 were found only in bacteria. Some of these domain-specific structural groups of Ni-CODHs, such as A-3, A-4, A-5, A-6, C-3, D-2, D-3, and D-4, were deeply branched in each clade (Figure 1 and Supplementary Table 2).

## Discussion

In this study, we performed a comprehensive analysis of an expanded Ni-CODH dataset focusing on their structures and genomic contexts in order to classify Ni-CODHs and their related proteins and predict their novel functions. The previous two comprehensive studies for classification of Ni-CODHs have only focused on specific genomic contexts (i.e., CODH/ACS, CODH/ECH, and CODH/CooF) or clades (Techtmann et al., 2012; Adam et al., 2018). Techtmann et al. (2012) focuses on CODH/ACS, CODH/ECH, and CODH/CooF, whereas Adam et al. (2018) focuses on CODH/ACS subunits in more detail. In particular, there are few descriptions in the functions of clades B and D Ni-CODHs and the structural diversity of Ni-CODHs. Our work provided more comprehensive and unbiased descriptions with larger dataset than these previous studies. Our phylogenetic analysis represented not only the previously reported six clades but also the novel clade G (Figure 1). Furthermore, our structure-based classification showed that the structural motifs of Ni-CODHs were more diverse and plastic than previously reported (Table 2 and Figures 2–4). Moreover, the motifs consisting of D-clusters were variable, even within the same clade. Additionally, the active-site motifs in well-characterized Ni-CODHs were not conserved in structural groups A-3, A-5, B-1, B-2, and B-3 or in any structural groups in clades C, D, and G.

Our structure-based classification also identified novel structural motifs of Ni-CODHs (Figure 2 and Supplementary Table 1). In particular, structural groups A-5 and C-4 have insertions of novel Cys motifs. The two Cys × 4 motifs in structural group A-5 were tandemly inserted in the same region of the E- and F-clusters, suggesting that additional two [4Fe-4S] clusters could conduct electron transfer. Conversely, one Cys × 4 motif in structural group C-4 were inserted between the Tyr residue and the His residue in the C-cluster predicted to be opposite side of the D-cluster on 3D structure, implying possible functions in protein-protein interaction or non-canonical electron transfer to the [FeFe] hydrogenase catalytic subunit (COG4624). In addition, structural groups C-1, D-3, E-3, and F-2 have another types of additional N-terminal Cys motifs in which the numbers of Cys residues were variable from two to four. The crystal structure of *R. rubrum* CooS indicates that the N-terminal Cys-containing region is disordered (Drennan et al., 2001). We speculated that these Cys-containing extensions might have a metallochaperone-like function. However, the structural and functional properties of these novel Cys motifs remain unknown.

Additionally, structural groups of B-1 and B-3 Ni-CODHs harbor His-rich extensions likely responsible for metal binding similar to the Ni chaperones CooJ and HypB (Figure 2 and Supplementary Table 1; Watt and Ludden, 1998; Olson and Maier, 2000). Our genomic context analysis also identified NitT/TauT-family ABC transporters as putative clade B Ni-CODH-associated proteins, which consist of three components: an ATP-binding cassette module, a transmembrane module, and a periplasmic solute-binding module (Figure 5 and Supplementary Tables 4, 5). We found that the periplasmic module is homologous to a periplasmic solute metal-binding protein from *Staphylococcus aureus* (PDB ID: 3UN6) (data not shown). The Ni-insertion machinery of Ni-CODHs involves a variety of metallochaperones (Merrouch et al., 2018). CooC is considered a major component in various organisms, whereas CooJ and CooT provide assistance in some organisms, such as *R. rubrum*, suggesting diverse Ni-insertion mechanisms in the maturation of Ni-CODHs. Therefore, we hypothesized that the ABC transporters import metal ions, such as Ni^2+^, and then the His-rich regions of structural groups B-1 and B-3 Ni-CODHs bind the metal ions and insert them into the catalytic sites of Ni-CODHs.

Our structural prediction analysis suggested that the active-site residues of structural group D-1 Ni-CODHs were similar to those of HCPs, although they were phylogenetically distant from each other (Figure 4). A previous study reported that a structural group D-1-mimicking mutant (C295E) of *C. hydrogenoformans* CooSII exhibits not only dramatically decreased Ni-binding and CO-oxidation activities but also increased hydroxylamine reductase activity (Inoue et al., 2013). Therefore, we speculate that structural group D-1 Ni-CODHs likely have similar structural and functional properties as HCPs as a result of convergent evolution. A genetic analysis of *Clostridium autoethanogenum* has also shown that structural group D-1 Ni-CODH is unable to compensate inactivation of AcsA (Liew et al., 2016). However, genetic analysis of *M. acetivorans* suggests potential involvement of structural group D-1 Ni-CODHs in CO oxidation (Rother et al., 2007). Therefore, the biochemical characterization of structural group D-1 Ni-CODHs is required to elucidate their catalytic activity.

Ni-CODH/FNOR represents a recently identified putative functional unit for CO oxidation (Whitham et al., 2015; Geelhoed et al., 2016); however, no biochemical study of this type of FNOR has been reported. Notably, structural groups C-1, C-2, D-3, and G-1 Ni-CODHs were related to FNOR and CooF according to our network analysis (Figure 5 and Supplementary Figures 2, 3). The genes for some structural group D-1, D-2, D-3, E-1, E-2, E-3, and F-1 Ni-CODHs also coexist with FNOR in their genomic contexts (Supplementary Tables 2, 5). The deletion mutant of a gene for structural group C-2 Ni-CODH adjacent to FNOR and CooF in *C. autoethanogenum* has exhibited growth deficiency in the presence of CO probably because of the excess reducing equivalent, suggesting its role in CO_2_ reduction (Liew et al., 2016). Conversely, transcriptomic analysis of *C. ljungdahlii* showed that expression of the gene cluster for Ni-CODH, comprising structural group C-1 Ni-CODH, FNOR, and CooF, is upregulated upon oxygen exposure, suggesting their use of CO as a source of electrons for oxygen detoxification (Whitham et al., 2015). Similar phenomenon was observed in structural group C-1 Ni-CODH from *C. acetobutylicum* although the genomic context was not conserved (Hillmann et al., 2009). Additionally, a study of *Geobacter sulfurreducens* growth in the presence of CO suggests that a gene cluster encoding structural group E-1 Ni-CODHs, FNOR, and CooF is likely responsible for CO oxidation (Geelhoed et al., 2016). Our analysis suggested that FNOR could be coupled with various structural groups of Ni-CODHs, and that the genes for FNOR are widespread across microbial genomes along with genes encoding Ni-CODHs, such as WLP or ECH.

Additionally, we showed that only structural groups E-1, F-1, and F-2 Ni-CODHs coexisted with the [NiFe] hydrogenase catalytic subunits based on their genomic contexts (Supplementary Tables 2, 5). These data imply a requirement of the complete structural motifs for Ni-CODH/ECH coupling. Conversely, all structural groups of Ni-CODHs in clades A, E, and F, except for structural groups A-3 and F-2, coexisted with the ACS β subunit regardless of their complete or incomplete structural motifs. However, it remains necessary to examine whether the structural groups harboring incomplete structural motifs are able to catalyze the CODH/ACS reaction.

Unveiling the specific interactions between Ni-CODH phylogeny and microbial physiology are quite challenging because of possible functional redundancy and horizontal gene transfer (Techtmann et al., 2012; Sant’Anna et al., 2015). Our dataset could provide some interactions between specific structural group and microbial physiology. It has been shown that carboxydotrophic organisms rely on clades A, E, and F Ni-CODHs for CO oxidizing activity (Matschiavelli et al., 2012; Techtmann et al., 2012; Sant’Anna et al., 2015). In our dataset, structural groups B-4 and D-3 Ni-CODHs were also specifically found in carboxydotrophic bacteria in addition to their clade F Ni-CODHs forming gene clusters with ACS or ECH (Supplementary Table 2). Structural group B-4 was found in carboxydtrophic genera *Moorella, Calderihabitans*, and *Thermanaeromonas* (a candidate carboxydtroph possessing a CODH/ECH gene cluster), whereas structural group D-3 was found in *Carboxydocella* and *Thermanaeromonas*. Interestingly, these structural groups possessed the complete motif for the C-cluster, suggesting possible roles for carboxydotrophic metabolism in these organisms. Conversely, methanogenic archaea depend on clade A Ni-CODHs for their acetoclastic methanogenesis (Gong et al., 2008; Techtmann et al., 2012). However, structural group C-3 was specifically found in methanogenic genera *Methanocaldococcus* and *Methanotorris* in addition to structural group A-1. We also found that structural group D-4 was conserved in anaerobic methane oxidizing genera *Candidatus Methanoperedens* with structural group A-2. These structural groups of Ni-CODHs might have possible roles in their unique metabolisms.

Our phylogenetic analysis also provided information clarifying the evolutionary history of Ni-CODHs. In Cdh-type Ni-CODHs (clade A), structural groups A-2 through A-6 not harboring the D-cluster were deeply branched, with structural groups A-2, A-4, A-5, and A-6 being more deeply branched than structural group A-3 not harboring the E- and F-clusters (Figures 1, 2). These data imply that the ancestor of Cdh-type Ni-CODHs did not likely harbor the D-cluster but rather the E- and F- clusters. This idea might be supported by a previous structural study of the *M. barkeri* ACS α subunit, which suggested that the E- and F-clusters are responsible for electron transfer to or from a ferredoxin-like protein rather than the D-cluster (Gong et al., 2008). In fact, structural groups A-2, A-4, A-5, and A-6 formed gene cluster with ACS subunits, suggesting possible involvements for WLP without D-cluster. Therefore, acquisition of the type I D-cluster in structural group A-1 might enable electron transfer to flavin adenine dinucleotide.

Moreover, in the CooS-type Ni-CODHs (clades B–G), structural groups harboring type II D-clusters were deeply branched in clades D and E, whereas structural group C-3 harboring the incomplete D-cluster was deeply branched in clade C, and structural group G-1 had a type III D-cluster. Regarding the catalytic site, structural groups B-4, D-2, D-3, and D-4 harboring the complete motif required to form the C-cluster were deeply branched, implying that mutations in the C-cluster motifs occurred after branching into each clade, and that the ancestor of clades B through F likely harbored the complete motif necessary to form the C-cluster. Conversely, structural group G-1 had a mutation in the second Cys residue of the C-cluster. These structural and evolutionary plasticities in CooS-type Ni-CODHs render it difficult to infer their ancestral form. Therefore, additional sequence information for clade G Ni-CODHs in particular is required to ascertain the ancestor of the CooS-type Ni-CODHs.

Previous studies suggested that the CODH/ACS complex likely existed in the last universal common ancestor (LUCA) (Weiss et al., 2016; Adam et al., 2018). However, it has been difficult to predict the Ni-CODHs that existed in the LUCA based on phylogenetic analysis of Ni-CODHs because of the horizontal gene transfer between domains. In our analysis, some of the domain-specific structural groups of Ni-CODHs were deeply branched in clades A, C, and D (Figure 1 and Supplementary Table 2). These data raise the possibilities that the ancestor of Ni-CODHs in each clade might have existed in the LUCA or might have been transferred between domains early after branching of each clade.

Conversely, we found a possible horizontal gene transfer across the domains in shallow branches of the tree. Only one archaeon *Methanosarcina horonobensis*, was found to possess a clade B Ni-CODH (structural group B-1) exhibiting high similarities (sequence identities >70%) to those from *Caldicoprobacter* and *Ruminiclostridium* genera. Furthermore, the structural group B-1 Ni-CODH from *M. horonobensis* was found in putative gene cluster of ABC transporters like other clade B Ni-CODHs. The three components of ABC transporters from *M. horonobensis* also showed high similarities (sequence identities >60%) to those from *Caldicoprobacter* and *Ruminiclostridium* genera. These results imply that there would be a variety of evolutionary histories of Ni-CODHs.

Overall, our expanded dataset and genomic context analysis revealed novel putative Ni-CODH-associated proteins and relationships among Ni-CODH structural features, genomic contexts, and taxonomies, suggesting unanticipated structural and functional diversity and plasticity. The enhanced understanding of biochemically uncharacterized Ni-CODHs, their associated protein functions, and Ni-CODH evolutionary history will likely facilitate future research in anaerobic microbe metabolism.

## Author Contributions

MI, TY, and YS conceived and designed the work. MI, IN, and TO constructed the datasets of Ni-CODHs and performed the phylogenetic analyses. MI performed the comparative sequence and structural prediction analyses. MI, IN, KO, and HO performed genomic context analysis. MI, TY, and YS wrote the paper with help from KO and HO.

## Conflict of Interest Statement

The authors declare that the research was conducted in the absence of any commercial or financial relationships that could be construed as a potential conflict of interest.
