# Supplementary material for: Structural and Phylogenetic Diversity of Anaerobic Carbon-Monoxide Dehydrogenases
Source: Front Microbiol. 2019 Jan 17;9:3353. doi: 10.3389/fmicb.2018.03353 (PMC6344411; doi:10.3389/fmicb.2018.03353)
Supplement: Supplementary file 6 [file Data_Sheet_1.PDF]

Supplementary Material

Structural and phylogenetic diversity of anaerobic carbon-monoxide dehydrogenases

Masao Inoue, Issei Nakamoto, Kimiho Omae, Tatsuki Oguro, Hiroyuki Ogata, Takashi Yoshida, Yoshihiko Sako*

* **Correspondence:** Yoshihiko Sako: sako@kais.kyoto-u.ac.jp

Supplementary Table 1. The Ni-CODHs protein dataset with structural features.
Presented as a separate MS Excel file.

Supplementary Table 2. The Ni-CODHs genome dataset with taxonomies and genomic contexts.
Presented as a separate MS Excel file.

Supplementary Table 3. A summary for structural prediction of structural groups of Ni-CODHs using SWISS-MODEL.
Presented as a separate MS Excel file.

Supplementary Table 4. The list of COGs in Ni-CODH-containing genomic contexts.
Presented as a separate MS Excel file.

Supplementary Table 5. A similarity matrix used for the network analysis.
Presented as a separate MS Excel file.

Supplementary Figures

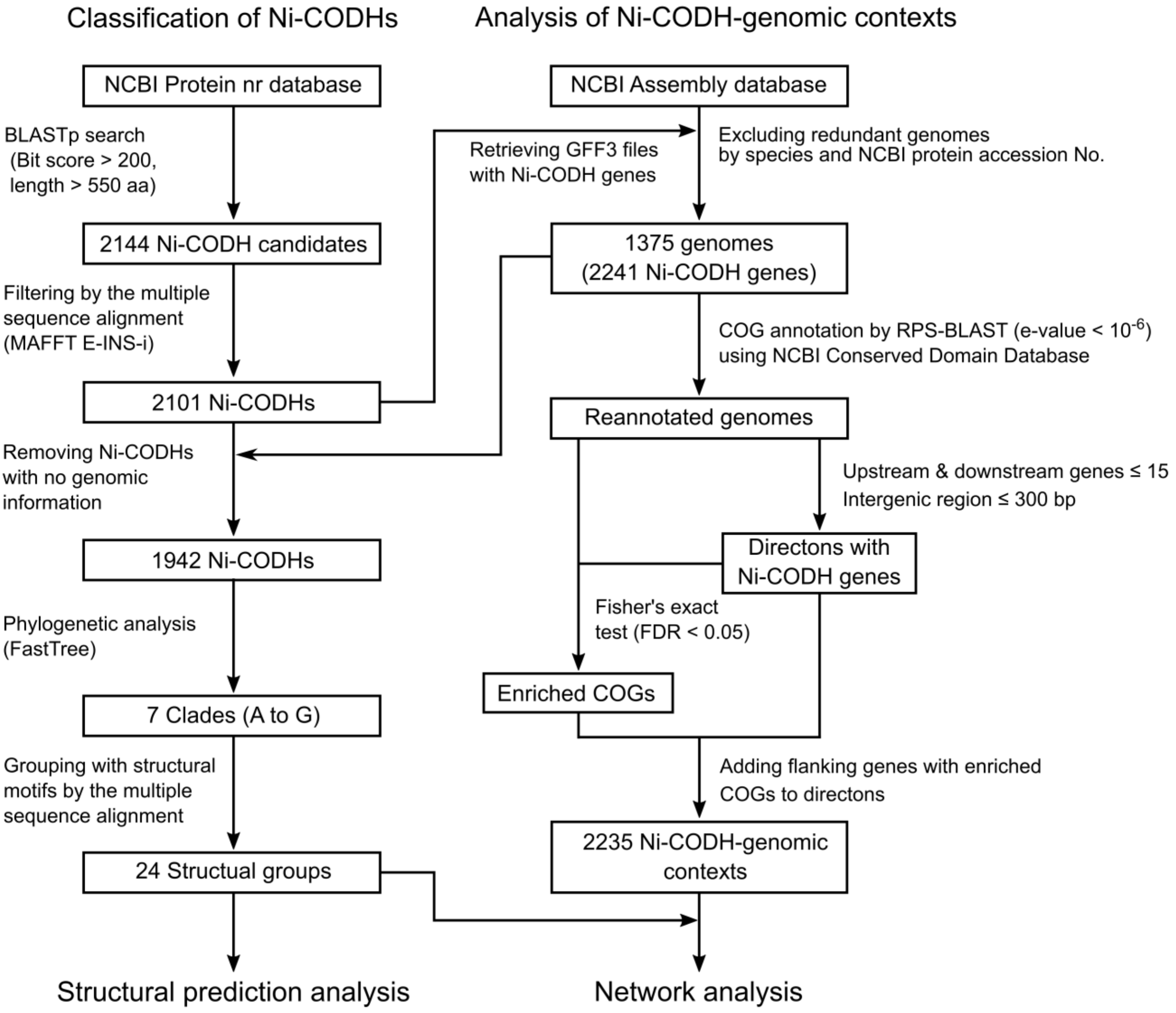

Supplementary Figure 1. A schematic representation of workflows for data analysis of Ni-CODH proteins and genomic contexts.

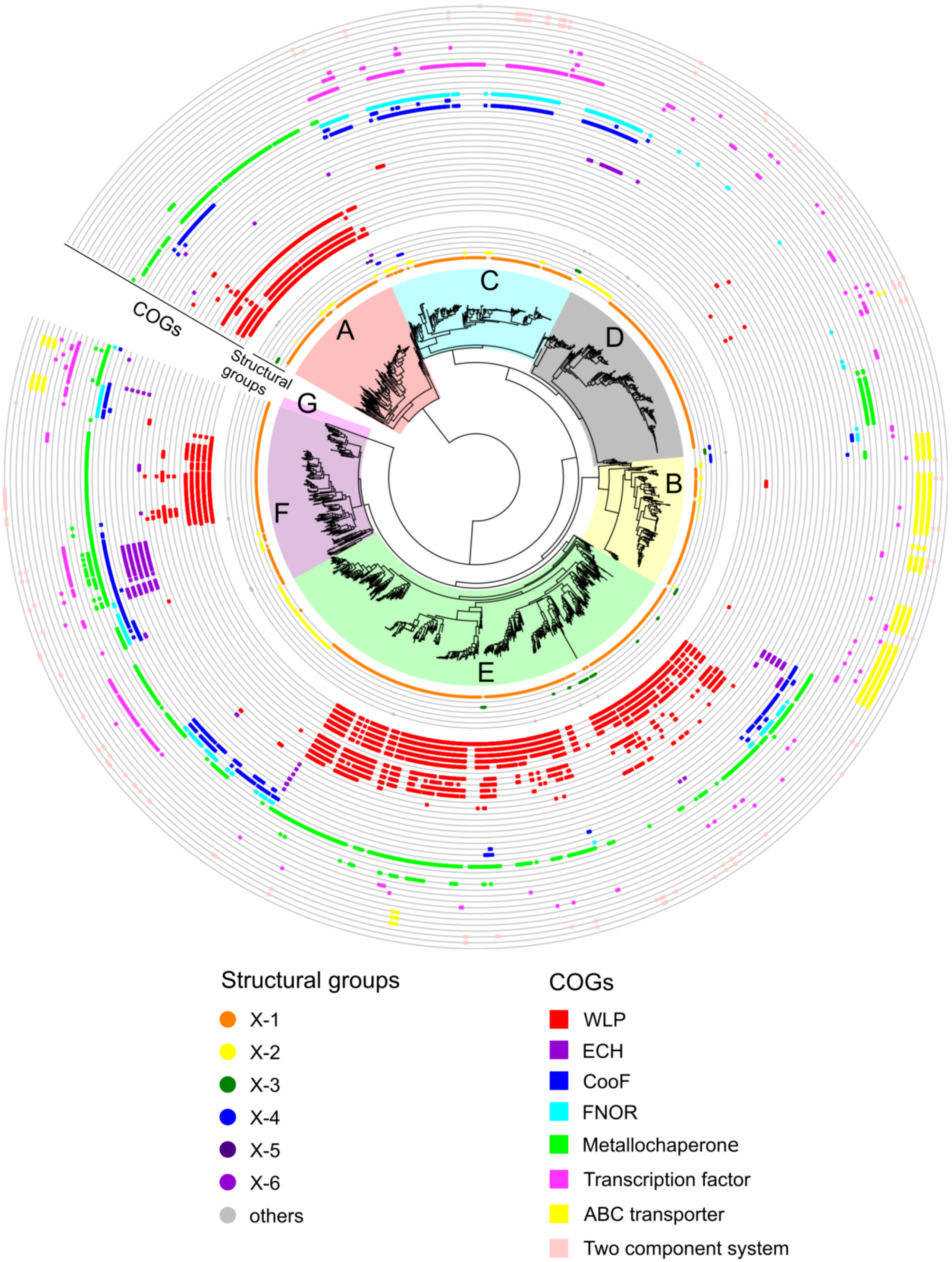
COGs
Structural groups
A
B
C
D
E
F
G
Structural groups
X-1
X-2
X-3
X-4
X-5
X-6
others
COGs
WLP
ECH
CooF
FNOR
Metallochaperone
Transcription factor
ABC transporter
Two component system

Supplementary Figure 2. Phylogenetic tree of Ni-CODHs with the information of structural groups and associated protein functions (related to Figures 1 and 5; Supplementary Figure 3). The structural groups of Ni-CODHs and the associated COGs are concentrically mapped on the phylogenetic tree and shown by filled circles and filled diamonds, respectively. Each clade is colored in similar way to Figure 1. The structural groups and COGs are colored according to their numbers (*i.e.* A-1, A-2, and so on) and functions as *inset*, respectively. The letter "X" in the *inset* corresponds to each clade.

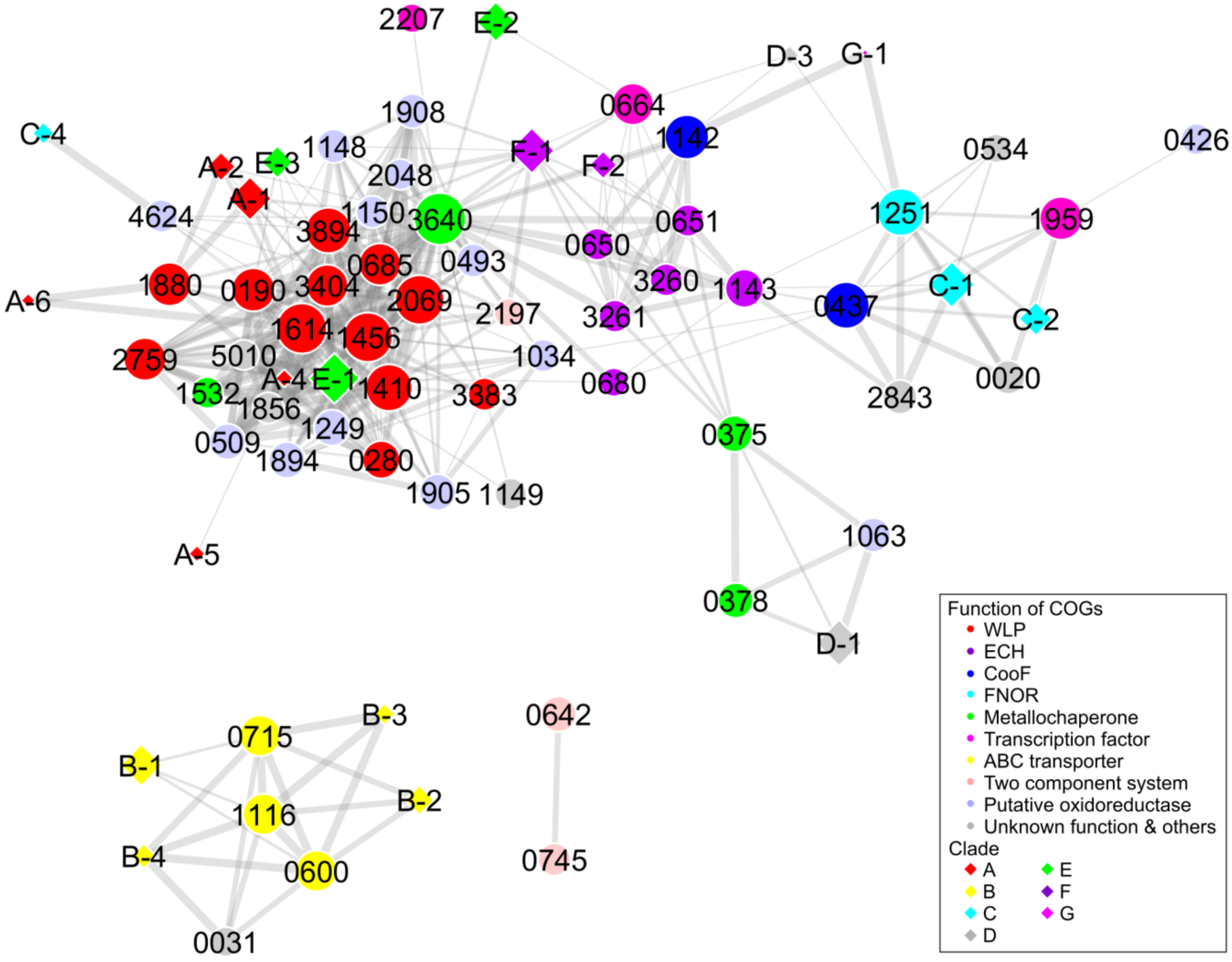

Supplementary Figure 3. Network analysis among Ni-CODH-related proteins and structural groups of Ni-CODHs (related to Figure 5). The network is represented by an unweighted force-directed layout and manually modified to avoid overlapping nodes. The width of the edges is scaled proportionally according to the Simpson coefficient. The size of the nodes is scaled proportionally according to the log of the number of genomic loci with each COG and structural group. The COGs and the structural groups of Ni-CODHs are shown by filled circles and filled diamonds, and coloured according to their functions and clades as *inset*, respectively.
